# How does workplace event criticality spur employees’ proactivity? The roles of work engagement and mindfulness

**DOI:** 10.3389/fpsyg.2022.976213

**Published:** 2022-11-07

**Authors:** Yi Zhang, Lifang Gao, Yuan Feng

**Affiliations:** ^1^Art Education Center, Southwestern University of Finance and Economics, Chengdu, China; ^2^School of Business Administration, Faculty of Business Administration, Southwestern University of Finance and Economics, Chengdu, China; ^3^School of Accounting, Southwestern University of Finance and Economics, Chengdu, China

**Keywords:** workplace event criticality, work engagement, mindfulness, proactive work behavior, event system theory

## Abstract

This paper aims to generate insights about *whether*, *how,* and *when* workplace event criticality spurs employee proactivity. We conducted multilevel analyses with a three-wave time-lagged survey of 179 employees and their 55 direct leaders in China to test our proposed model. The findings indicate that workplace event criticality is conducive to stimulating proactive work behavior through improving employee engagement. Further, employee mindfulness amplifies the positive relationship between workplace event criticality and work engagement. Despite the increasingly unavoidable influence of events in the workplace on employee proactivity, empirical research around the relationship and its underlying mechanism has been rather sparse. Our event-oriented research advances this knowledge by unpacking the salient motivating role of workplace events’ criticality in employee work engagement and proactivity. It also increases our understanding by illustrating that employee mindfulness will amplify and intensify the motivational potential of workplace event criticality for work engagement.

## Introduction

As the business environment becomes increasingly uncertain and dynamic, unexpected happenings or events have emerged at every organizational level. Defined as “occurrences that interrupt the routines of organizational life and prompt-controlled information processing” (Morgeson and DeRue, 2006: 273), workplace events will exert undeniable influence over employees (Chen et al., 2021; Liu et al., 2021). Examples of workplace events include introducing new manufacturing equipment, implementing a new performance appraisal system, and unexpected promotions. According to event system theory, these events may be salient in shaping employee behavior (Morgeson et al., 2015). However, a large body of extant research has employed a feature-oriented perspective by focusing on the continuous and stable features while neglecting the discrete and unpredictable events that penetrated the current dynamic and uncertain context (Chen et al., 2021; Liu et al., 2021). In a comprehensive review of studies considering the importance of context, Johns (2017, p. 584) pointed out that “if there has been a deficit in contextual theorizing, it is most apparent in a basic lack of theories that treat discrete events as contexts.” To fill this gap, we consider event system theory a valid basis for quantifying the impact of workplace events and investigating whether and how workplace event characteristics impact employees’ workplace behaviors. This endeavor contributes to the theoretical and empirical understanding of workplace events.

Studies have claimed that the more salient the workplace event, the more likely it will be to stimulate change and/or create new behaviors and features (Crawford et al., 2019). In line with these studies, we adopt the concept of event strength proposed by Morgeson et al. (2015) and focus on the effects of one key event’s characteristic (i.e., event criticality) on employee behavior. Workplace event criticality reflects “the degree to which an event is important, essential or a priority” (Morgeson and DeRue, 2006: 273). Criticality helps a workplace event stand out, triggering cognitive, psychological, and behavioral changes. When employees consider workplace events critical, they are motivated to display higher initiative in their work to improve their current circumstances rather than passively adapting to the present conditions (Liu et al., 2018). For instance, when an organization implements a new performance appraisal system, employees regarding this event as critical will actively adjust to new work conditions. However, to our knowledge, few empirical studies have investigated the impacts of workplace event criticality on employee proactivity. We contend that this lack of research is an oversight. To bridge this gap, we propose that critical happenings have great motivational potential to inspire employees’ proactive work behaviors, focusing on self-initiated and anticipatory action in uncertain contexts (Parker et al., 2006).

By integrating the theory of engagement and event system theory, we introduce work engagement as an avenue to understand the psychological process underlying the relationship between workplace event criticality and its eventual proactive work behavior (Kahn, 1990; Christian et al., 2011). Work engagement, defined as “a positive, fulfilling, work-related state of mind that is characterized by vigor, dedication, and absorption” (Schaufeli et al., 2002, p. 465), in nature, is a motivational concept (Bledow et al., 2011). Faced with a critical workplace event, employees are motivated to self-invest their personal resources (e.g., energy and time) for personal growth and goal attainment (Christian et al., 2011). Hence, we presume that an employee may feel energetic in critical workplace events, which will affect the employee’s proactive work behavior.

Event system theory suggests that “when examining events, scholars should not ignore the critical role of [one’s internal] features but should construct an integrative theory-building approach that examines the ways features and events jointly or independently affect entities. This may enable the development of more fine-grained organizational theories, enhancing their explanatory power and impact” (Morgeson et al., 2015, p. 530). Therefore, to further leverage the contingency perspective on event characteristics, we also examine the moderating role of employees’ mindfulness on the relationship between workplace event criticality and work engagement. Mindfulness, “a receptive attention to and awareness of present events and experience” (Brown et al., 2007a, p.212), reflects the variations in an individual’s quality of consciousness (Brown et al., 2007a). Recent research has found that mindfulness can change the way individuals perceive and process the information conveyed by external events and occurrences and thus influence employees’ psychological and behavioral reactions (Brown et al., 2007a). Employee mindfulness may act as an amplifier and enhance the influence of event characteristics on work engagement, given the increased attention and consciousness to manage the uncertainty associated with mindful employees. This would be crucial to the correspondence for unpredictable events that need immediate attention (Brown et al., 2007a). Thus, the impact of workplace event criticality on work engagement should be more substantial for mindful employees.

**Figure 1:**
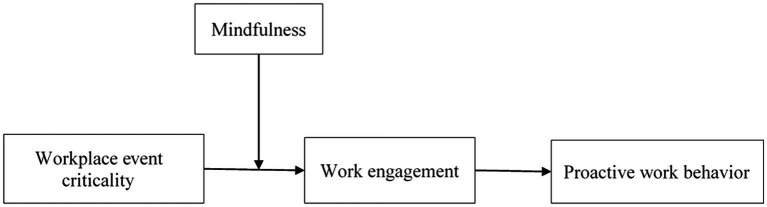
Conceptual research model.

Overall, this study contributes to the literature in three aspects. First, we employ an event-oriented perspective and empirically examine whether workplace event criticality is conducive to facilitating proactive work behavior with a multi-source and multi-wave field study. This research endeavor provides new theoretical insights into organizational behaviors by focusing on the impact of unpredictable happenings, rather than routinized organizational life, on employees’ behavioral responses. Second, this study extends event system theory by integrating the theory of engagement and introducing work engagement as a unique mechanism that helps to explain how employees react and respond to critical events at work. In doing so, we shed light on the psychological mechanism underlying how critical workplace events transfer their influence onto work behavior. We also reveal the motivational potential of critical workplace events for stimulating employee engagement and proactivity. Third, we establish an integrative model by examining how employees’ internal stable features (i.e., mindfulness) interact with the external dynamic workplace events and jointly shape their responses. This enables the development of a more fine-grained theoretical model and expands our understanding of which type of employees the motivational potential of critical events can be attenuated or accentuated. Our theoretical model is depicted in Figure 1.

## Literature review and hypotheses development

### Event system theory

This study mainly used event system theory (EST) to establish theoretical grounds for accounting for workplace events’ impact (Morgeson et al., 2015). According to EST, events become salient when they are novel, disruptive, and critical. Event criticality reflects “the degree to which an event [perceived by employees] is important, essential or a priority” to employees (Morgeson and DeRue, 2006, p. 273). Event novelty reflects the extent to which an event distinguishes itself from current and past behaviors, features, and events, therefore emerging as a novel or surprising phenomenon (Morgeson, 2005). Event disruption reflects how an event prevents employees from getting their work done and impedes their routines (Morgeson et al., 2015). According to EST, although each characteristic represents different aspects of an event, they can function independently (e.g., Morgeson and DeRue, 2006; Chen et al., 2021). For instance, Chen et al. (2021) examined the role of workplace event novelty in fueling employee improvisation. In addition, Morgeson and DeRue (2006) showed that the more critical events are, the more disturbances such events will cause to teams. Hence, it is possible that even one characteristic can yield a strong enough event to inspire changes and the creation of behaviors.

In our research, we focus on the impact of workplace event criticality. Criticality can help a workplace event stand out and triggers in-depth interpretation, variance, and psychological processes. As Morgeson et al. (2015, p. 521) emphasize, “the more critical the event, the more likely it will be seen as salient and require unusual attention and action.” In other words, highly critical workplace events demand attention and motivate employees to devote more of their resources on a priority basis to respond to the event (Liu et al., 2018). For instance, Morgeson and DeRue (2006) highlight that highly critical events in teams would become the central focus of teams until the events are resolved.

In addition, given that employees’ resources and energies are limited, they tend to invest a lot of personal resources on a priority basis to respond to events bringing high criticality and importance for the long-term success of employees (Craft and Leake, 2002). Compared with uncritical trifles, events with high criticality signify a great opportune time for personal development and career success. Hence, highly critical events can motivate employees to invest more resources toward addressing them. In this regard, exploring the impact of workplace event criticality is of high importance. Event system scholars posit that psychological processes exist that transpire between workplace events happening and call for researchers to integrate other theories to examine the underlying psychological process between event happenings and the behavioral outcomes. Therefore, we combine Kahn’s engagement theory into event system theory to explore the underlying mechanism of how event criticality impacts employees’ proactive behavior.

### The integration of Kahn’s engagement theory with event system theory

According to Kahn’s engagement theory, work context influences employee work engagement and drives positive behavioral outcomes, such as proactive work behavior, job involvement, and job performance (Kahn, 1990, 1992; Christian et al., 2011). Existing research mainly focuses on the impact of some relatively stable and continuous aspects of the work context on employee work engagement, such as task characteristics (Salanova et al., 2005), role characteristics (Christian et al., 2011), and management styles (Tims et al., 2011). This stream of research emphasizes the motivational potential embedded in some stable aspects of the work context, like job characteristics (Hackman, 1980; Oldham and Hackman, 1981), and has provided a great deal of insight into their effects on engagement.

In this new VUCA world (VUCA stands for volatile-uncertain-complex-ambiguous), more and more unpredictable events happen at various organizational levels (Bennett and Lemoine, 2014). Events reflect discontinuous and discrete happenings that diverge from the work context’s stable or routine features. These workplace events injected strong vitality and also disturbance into organizational life. Yet, existing studies have not paid sufficient attention to the fact that discrete events are a critical component of the work context (Johns, 2017). According to Bledow et al. (2011), employees’ level of work engagement is impacted by many aspects (e.g., stable and dynamic) of the work context in which employees are embedded. As a result, it is necessary to include the event in the work context and explore whether event characteristics (e.g., criticality) influence employee engagement. Therefore, in this study, we consider employee work engagement to be an important mediator in transmitting the impact of unanticipated workplace events on employees’ proactive behaviors. Below we will detail the mediating role of work engagement.

### The mediating role of work engagement

Building on event system theory, we first contend that workplace event criticality may motivate employee work engagement. Salanova and Schaufeli (2008) define work engagement as a positive, fulfilling, affective-motivational state of employee well-being characterized by vigor, dedication, and absorption. Specifically, vigor refers to working in a highly energetic state; Dedication refers to being strongly involved with work; and absorption refers to being fully concentrated and happily engrossed at work (Wang et al., 2015). In the following discussion, we elaborate on the impact of workplace event criticality on work engagement in these three aspects.

First, workplace event criticality can motivate employees’ vigor. When an individual faces an unfamiliar task or situation or is exposed to high criticality/significance stimuli, they will have an increasingly strong sense of emotional arousal (Scott, 1966). Similarly, when these unpredicted happenings are perceived as critical, employees tend to treat them as stimuli, which may create a heightened sense of arousal (Wang et al., 2015). This heightened arousal, in turn, galvanizes employees to feel vigorous in addressing the events (DeRue and Wellman, 2009).

Second, workplace event criticality is an important determinant of dedication expected to inspire employees’ willingness to work harder (Hackman and Oldham, 1974, 1976; Grant, 2008). When employees view workplace events as critical to their work, they feel strong personal responsibility and become motivated to self-invest their resources (e.g., time and energy) into their work (Macey and Schneider, 2008; Christian et al., 2011). This contention is corroborated by Crawford et al. (2019), who posited that the more critical the event is, the more likely employees are to invest increasing effort into their tasks.

Third, workplace event criticality has a positive effect on absorption. Scholars have suggested that critical workplace events disrupt the *status quo* and cause disturbance to the current situation. This, in turn, requires employees to pay additional attention to these events (Morgeson and DeRue, 2006; Morgeson et al., 2015). Accordingly, when encountering critical workplace events, employees tend to concentrate fully on the emerging problem and ignore uncritical trifles. At the same time, critical workplace events are usually time-sensitive, and employees must respond to them promptly. In such urgent situations, employees must remain fully attentive and focused on dealing with the problems within limited time constraints (Morgeson, 2005). To summarize, workplace event criticality is essential to vigor, dedication, and absorption, which are reflected in work engagement. Thus, we hypothesize the following:

*H1:* Workplace event criticality is positively related to employee work engagement.

We posit that work engagement can serve as a motivational process that underpins employee proactive work behavior. In such a process, employees feel energetic at work and have a strong sense of identity with their organization. This affection spurs proactive work behavior (Halbesleben and Wheeler, 2008). Specifically, work engagement may facilitate employees’ proactivity in three ways.

First, engaged employees tend to feel more vigorous at work. Such a feeling could motivate them to proactively invest more effort into dealing with problems in the workplace (Sonnentag, 2003). Meanwhile, work engagement could encourage employees to express attachment to their organization and spur employee proactivity (Zhou and George, 2001; Crawford et al., 2019). Second, work engagement implies that employees are enthusiastic at work and dedicated to their jobs (Salanova and Schaufeli, 2008). This means engaged employees tend to be optimistic, leading them to engage in proactive behavior at work (Salanova and Schaufeli, 2008; Parker et al., 2010). Third, engaged employees are more likely to find their work exciting and meaningful and take pride in it (Parker and Griffin, 2011; Pompuang et al., 2019). Research has shown that employees who have this affection for their job tend to show personal initiative at work and bring about changes to improve their current work situation (Sonnentag, 2003; Wang et al., 2015).

Combined with H1, employees’ work engagement levels increase when they perceive workplace events as critical. These heightened work engagement levels, in turn, fosters their proactive work behavior. Taken together, we hypothesize the following:

*H2:* Workplace event criticality has a positive and indirect effect on employee proactive work behavior through employee work engagement.

### The moderating effects of mindfulness

Event system theory and research suggest that scholars adopt an integrative theory-building approach to examining how an individual’s internal features, combined with the external events they experiences, may jointly affect that person’s reactions (e.g., Chen et al., 2021; Liu et al., 2021). For instance, in work-life boundary research, Crawford et al. (2019) showed that work-life shock events could interact with the internal attributes of a dual-earner couple to affect their subsequent psychological and behavioral responses significantly. Likewise, in the organizational context, we suggest that workplace event criticality could interact with employee features (e.g., mindfulness) to impact employee work engagement.

Work engagement is the simultaneous employment and expression of a person’s “preferred self” in a work role (Rich et al., 2010). Thus, the extent to which an employee is mindful plays a critical moderating role in determining their personal engagement when coping with critical events in the workplace. Mindfulness as a quality of consciousness is characterized by receptive attention to and awareness of current events and experiences without evaluation, judgment, or cognitive filters (Brown and Ryan, 2003; Glomb et al., 2011). Mindfulness can help people become more attentive in their existing activities. This is conducive to keeping employees interested, immersed, and involved in their work (Brown et al., 2007a; Long and Christian, 2015). Therefore, mindfulness is an important individual feature influencing employee engagement when responding to critical workplace events. In response to the call to develop an integrative model that examines the potential interaction between an event’s characteristics and an employee’s dispositions (Morgeson et al., 2015), our study investigates the interactive effect of workplace event criticality and employee mindfulness on employee work engagement.

We argue that the positive relationship between workplace event criticality and work engagement can be accentuated when employees have a higher level of mindfulness. Employees with a higher level of mindfulness are more likely to engage with work through focused attention to their current events and experiences. As illustrated above, coping with workplace events with high criticality requires an employee to devote more of their attentional resources to solving emerging problems (Morgeson and DeRue, 2006). Mindfulness entails focusing the employee’s attention on the events of the moment rather than becoming preoccupied with thoughts about the past or future (Brown and Ryan, 2003; Good et al., 2016). When workplace events are perceived as critical, mindful employees will focus their attention on them and respond to them in a more attentive and immersed manner (Leroy et al., 2013; Dane and Brummel, 2014). As such, when a mindful employee encounters critical workplace events, they are more likely to become more engaged by focusing on addressing the events.

In addition, mindful employees tend to have more flexible awareness and attention (Brown et al., 2007a; Glomb et al., 2011). This capacity means that employees can be mindful of all that is currently salient and be mindful of something particular, such as focusing on a stimulus or phenomenon according to the circumstances (Good et al., 2016). Criticality helps a workplace event stand out and demand attention. Based on this conceptual flexibility, when experiencing critical workplace events, mindful employees can disengage from distracting thoughts and emotions and sustain engagement with these events (Long and Christian, 2015). Conversely, mind*less* individuals cannot efficiently control their attention and will allocate additional attention resources to deal with off-task thoughts or activities (Cahn and Polich, 2009). This, in turn, hampers employees’ engagement processes. Taken together, we hypothesize that:

*H3:* Mindfulness moderates the relationship between workplace event criticality and work engagement, such that the relationship will be stronger for employees with a higher level of mindfulness.

We have argued that workplace event criticality influence work engagement (H1). Furthermore, we proposed that work engagement acts as the mediator by which event criticality link to employee proactive work behavior (H2). Then, we posited that mindfulness presents an important moderator of the relationship between workplace event criticality and work engagement (H3). These relationships reveal a moderated mediation model, as displayed in Figure 1. To fully capture all relationships of this proposed model, we formulate an additional hypothesis indicating the conditional indirect effect of workplace event criticality on proactive work behavior through work engagement, such that the impact is more pronounced for employees with a higher level of mindfulness.

*H4:* Mindfulness moderates the indirect effects of workplace event criticality on proactive work behavior via work engagement, such that the indirect effects will be stronger for employees with a higher level of mindfulness.

## Materials and methods

### Sample and procedures

We collected three waves of multi-source data from full-time employees and their leaders in 60 different teams of five service-oriented companies in China. These companies are from industries including finance and securities, information technology, education, consulting, and food services. Following the principle of resource availability, we generated a company list from the service industry through the personal contacts of one of the authors. We limit the companies to service industries because employees in service-oriented companies encounter and deal with various events in their daily work, which suits the research objectives of this paper. In addition, companies’ selection was also based on geographic proximity; they are all located in a provincial capital city in western China. Participants were selected based on voluntary participation, and we informed the participants of the research purpose, survey procedure, response confidentiality, and incentives. Finally, 240 employees and 60 leaders from 60 teams participated. The teams operated in areas including research and development (R&D), technical support, customer service, and marketing. During data collection, the participants completed their surveys and returned them to the research assistant, who then combined the questionnaires with those completed by leaders and followers from the same team to create a matched-pair sample. Each participant received cash remuneration (RMB 100) as motivation after completing all the surveys. Participants provide their ratings on paper-and-pencil questionnaires.

The data were collected in three waves to minimize the common method bias and better test our study’s proposed causal relationships (Podsakoff et al., 2003). At Time 1, we invited 240 employees to rate the workplace event criticality, mindfulness, demographics, etc. Three weeks later (Time 2), employee participants were asked to rate their work engagement. Finally, 3 weeks after the second survey wave (Time 3), we invited 60 leaders to rate employees’ proactive work behavior and provide demographics.

We finally received valid and matched responses from 179 employees (response rate = 74.6%) nested within 55 leaders (response rate = 91.7%). Among these 179 employees, 85.5% were below 35 years old, 44.7% had bachelor’s degrees, and 41.9% were men. On average, an employee’s organizational tenure was 4.08 years (*SD* = 3.85). Among the leaders, 79.9% were below 40 years old, 54.2% had bachelor’s degrees, and 40.8% were men. On average, their organizational tenure was 8.18 years (*SD* = 4.24). We compared the demographic variables between the 240 observations collected at Time 1 and the 179 observations collected at Time 3 and found no significant variance between these two samples on all demographic variables through one-way ANOVA (age: *F* = 0.60, *p* = 0.81; Gender: *F* = 0.14, *p* = 0.71; Education: *F* = 0.02, *p* = 0.88; Tenure: *F* = 0.15; *p* = 0.70), which indicated that there was no sample attrition bias.

### Measures

The survey instrument was administered in Chinese. Since all the measures used in our study were initially developed in English, we invited one bilingual organizational behavior scholar to employ the translation and back-translation procedure to translate the measures into Chinese to achieve linguistic equivalence (Brislin, 1986). To ensure the item clarity, another two bilingual organizational behavior scholars and a group of Ph.D. students were invited to review the translation. We made minor changes based on their feedback. Five-point Likert scales (1 = strongly disagree and 5 = strongly agree) were used unless otherwise specified.

#### Workplace event criticality

We adopted the two-phrase process developed by Morgeson (2005) to collect data on workplace event criticality. Initially, the employees were invited to recall an event they had experienced in the workplace over the past 1 or 2 months (Chen et al., 2021). Since the event’s valence (i.e., positive, neutral, and negative) will confound the implications of event criticality, we only focused on positive events in this study (Wang et al., 2020). They recalled a range of positive events in this phase, some of which are illustrated in Table 1. Each employee was then asked to rate the degree of event criticality for the single event of their choice. Workplace event criticality was measured using a three-item scale from Morgeson and DeRue (2006) with a five-point Likert scale (1 = *much smaller extent* and 5 = *much larger extent*). Sample items included: “To what extent was this event critical for my long-term success” (*α* = 0.82).

**Table 1:** Examples of events.

Receiving a compliment from the leader
Receiving testimonials from the customers
Surpass the production target
Unexpected promotions
Adopting advanced technologies (e.g., robotic hand) to streamline of workflow processes
Launching new products into the market
Joining a new team
Becoming a full employee after internship assessment

#### Employee mindfulness

Employees were invited to assess their mindfulness with the 15-item Mindful Attention and Awareness Scale (MAAS) developed by Brown and Ryan (2003). Sample items included “I find it difficult to stay focused on what’s happening in the present.” The scale was reverse-coded to facilitate interpretation. Higher values in this study represented higher mindfulness (*α* = 0.89).

#### Work engagement

The employees were asked to rate their work engagement while considering their experienced workplace events reported at Time 1 using the Utrecht Work Engagement Scale from Schaufeli et al. (2006). Each dimension of work engagement was measured by three items. Sample items included “At my work, I feel bursting with energy” (vigor), “I am enthusiastic about my job” (dedication), and “I am immersed in my work” (absorption). We conducted a second-order factor analysis to check the homogeneity of the three dimensions. The results showed an acceptable fit to the data: *χ*^2^_[24]_ = 56.85, the Tucker-Lewis Index (TLI) = 0.91, the comparative fit Index (CFI) = 0.94, the root mean square error of approximation (RMSEA) = 0.09, and the standardized root mean square residual (SRMR) = 0.04 (*α* = 0.88).

#### Proactive work behavior

The above explainable variables are measured with self-reported scales. To minimize the common method bias, we did not use the same respondents as the source for obtaining proactivity data (Podsakoff and Todor, 1985; Jakobsen and Jensen, 2015; Tehseen et al., 2017). Instead of self-reported measures, the supervisors were invited to rate their subordinate’s proactive work behavior over the past 3 weeks by adopting the eight-item scale from Parker et al. (2006). Sample items included “Trying to find out why the product/service quality and/or level of performance decline” (*α* = 0.90).

#### Control variables

In line with prior research, we controlled for employee *age*, *gender*, *education*, *organizational tenure*, *team size*, and *team tenure* as existing research suggests that these variables affect employee engagement and proactive behavior performance (Sonnentag, 2003; Bakker and Schaufeli, 2008; Grant et al., 2009; Tang et al., 2020; Song et al., 2022). In addition, *event novelty and disruption*, representing other two critical aspects of event characteristics, may exert a confounding effect on employee behavioral responses (Morgeson et al., 2015). Thus, we also controlled for workplace event novelty and disruption. Employees were invited to measure these two event characteristics with a 5-point Likert scale ranging from 1 = *much smaller extent* to 5 = *much larger extent* at Time 1. Workplace event novelty was rated using the four-item scale from Morgeson (2005). Sample items included “To what extent there is a clear, known way to respond to this event” (reserved coded; *α* = 0.84). Workplace event disruption was rated using the four-item scale developed by Morgeson (2005). Sample items included “To what extent this event disrupts my ability to get my work done” (*α* = 0.85).

### Analytic strategy

Given that the employees were nested within teams, we conducted two-level modeling with maximum likelihood estimation with robust standard errors (MLR) to account for data non-independence in *Mplus* 7.4 (Muthén and Muthén, 2019). In line with previous studies, we grand mean-centered workplace event criticality and employee mindfulness when creating the interaction term to prevent multicollinearity (Hofmann and Gavin, 1998; Liu et al., 2021). To test the mediating hypothesis, we employed the product of coefficients proposed by Bauer et al. (2006) to compute the indirect effect (i.e., workplace event criticality→work engagement→proactive work behavior). Further, we tested its significance through a Monte Carlo simulation with 20,000 replications to generate the 95% confidence intervals (95% CI) in *R* 3.5. To test the moderated mediation hypothesis, we compute the conditional indirect effects at low (−1 *SD*) and high (+1 *SD*) values of the moderator (see also: Baer et al., 2015; Matta et al., 2017). If the CI for the conditional indirect effects difference excluded zero, then the moderated indirect effects were significant. *Pseudo*-*R*^2^ was calculated using the formula Snijders and Bosker (1999) proposed to evaluate the amount of variances in the mediator and dependent variable explained by the predictors.

## Results

### Confirmatory factor analyses

We conducted confirmatory factor analyses (CFAs) to test the distinctiveness of the four focal constructs (i.e., workplace event criticality, mindfulness, work engagement, and proactive work behavior). We used the balanced item parceling technique to optimize the sample size to parameter ratio (Little et al., 2002). Specifically, we keep the original theoretical structure for the multi-dimensional construct and create one parcel for each dimension. For the unidimensional construct, we created three parcels per construct. The four-factor model yielded an acceptable fit to the data: *χ*^2^_(48)_ = 73.93, *p* < 0.01; TLI = 0.96, CFI = 0.97, RMSEA = 0.06, SRMR = 0.06. This model fits the data significantly better than alternative models, including all of the three-factor models that combined two of the five constructs [65.59 ≤ △χ^2^_(3)_ ≤ 322.55], and single-factor model [△χ^2^_(6)_ = 452.52].

### Hypotheses testing

Table 2 reports the means, standard deviations, and zero-order correlations of all study variables.

**Table 2:** Means, standard deviations, and correlations.

Variables	Mean (*SD*)	1	2	3	4	5	6	7	8	9	10	11	12
1. Team size (time 3, level 2)	8.15 (4.55)	--											
2. Team age (time 3, level 2)	6.54 (3.53)	−0.10	--										
3. Age^a^ (time 1, level 1)	2.43 (1.21)	−0.13	0.03	--									
4. Gender^b^ (time 1, level 1)	0.58 (0.49)	0.17^*^	0.11	−0.16	--								
5. Education^c^ (time 1, level 1)	3.43 (0.60)	−0.04	−0.08	−0.07	0.00	--							
6. Work tenure (time 1, level 1)	4.08 (3.85)	−0.06	0.02	0.59^**^	−0.10	0.12	--						
7. Event novelty (time 1, level 1)	2.08 (0.63)	0.03	0.09	−0.12	0.15^*^	−0.12	−0.08	**(0.84)**					
8. Event disruption (time 1, level 1)	2.70 (0.87)	0.07	−0.01	−0.02	0.00	−0.09	0.04	0.12	**(0.85)**				
9. Event criticality (time 1, level 1)	4.04 (0.69)	0.13	0.16^*^	0.16^*^	0.01	0.03	0.17^*^	−0.43^**^	0.14	**(0.82)**			
10. Mindfulness (time 1, level 1)	3.59 (0.58)	0.05	0.13	0.04	0.13	0.08	0.04	−0.05	−0.31^**^	0.05	**(0.89)**		
11. Work engagement (time 2, level 1)	3.51 (0.56)	0.14	−0.05	−0.07	−0.05	0.08	−0.04	−0.12	0.08	0.25^**^	−0.14	**(0.88)**	
12. Proactive work behavior (time 3, level 1)	3.71 (0.63)	0.01	−0.23^**^	0.03	−0.17^*^	0.09	0.00	−0.15	0.03	0.12	−0.09	0.40^**^	**(0.90)**

*N* = 179 at the employee level (level 1); *N* = 55 at the team level (level 2); Organizational tenure and team tenure are measured in years; Consistency reliability appears along the diagonal in the brackets.

a Dummy-coded: 1 for less than 26, 2 for 26–30, 3 for 31–35, 4 for 36–40, 5 for 41–45, 6 for 46–50, 7 for 51–55, 8 for 56–60, and 9 for 60 or older.

b Dummy-coded: 0 for male, 1 for female.

c Dummy-coded: 1 for middle school and below, 2 for high school, 3 for an associate degree, 4 for a bachelor’s degree, and 5 for a master’s degree or above.

* *p* < 0.05.

** *p* < 0.01 (two-tailed).

**Figure 2:**
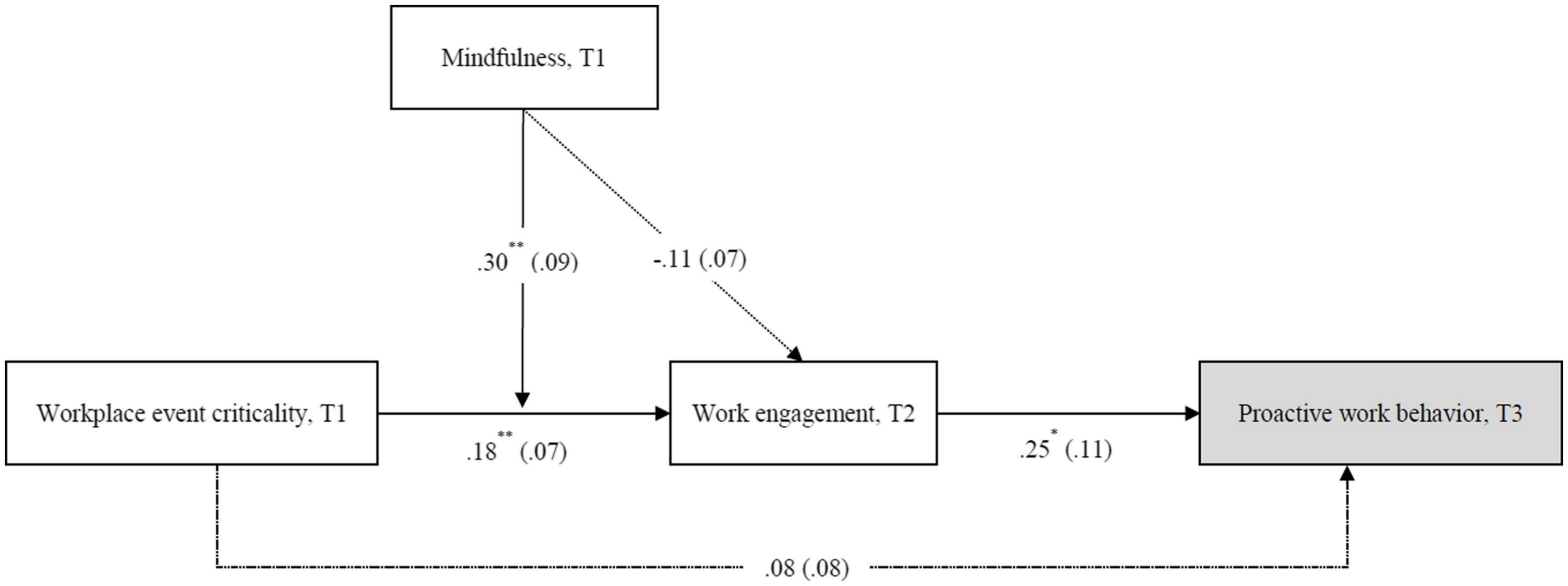
Multilevel path analyses results. Unstandardized coefficients are reported, and standard errors are shown in parentheses. Dashed lines represent paths that are not hypothesized. The grey variable represents the supervisor-rate variable. Employee level, *N* = 179; Team level, *N* = 55. T1 = time 1, T2 = time 2, and T3 = time 3. For clarity, control variables are not shown in the Figure. ^*^ p < 0.05, ^**^ *p* < 0.01 (two-tailed).

Figure 2 shows the multilevel path analysis results. H1 predicted that workplace event criticality would have a positive effect on employee work engagement. The results indicated that workplace event criticality was significantly and positively related to employee work engagement (*β* = 0.19, *p* < 0.01), yielding support to H1.

H2 predicted that workplace event criticality would have a positive indirect effect on employee proactive work behavior through employee work engagement. Our results showed that the direct effect of employee work engagement on employee proactive work behavior was also positive and significant (*β* = 0.25, *p* < 0.05). Additionally, the indirect effect of workplace event criticality on proactive work behavior *via* work engagement was positive and significant (*indirect effect* = 0.05, 95% CI [0.003, 0.11], excluding 0). H2 was thus supported.

**Figure 3:**
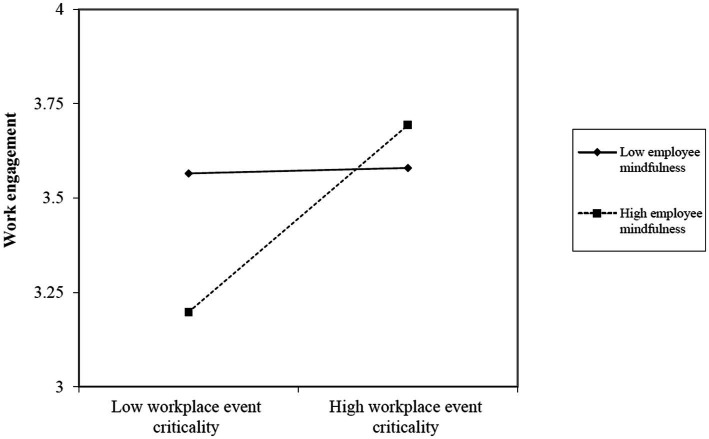
Interactive effects of workplace event criticality and employee mindfulness on work engagement.

**Figure 4:**
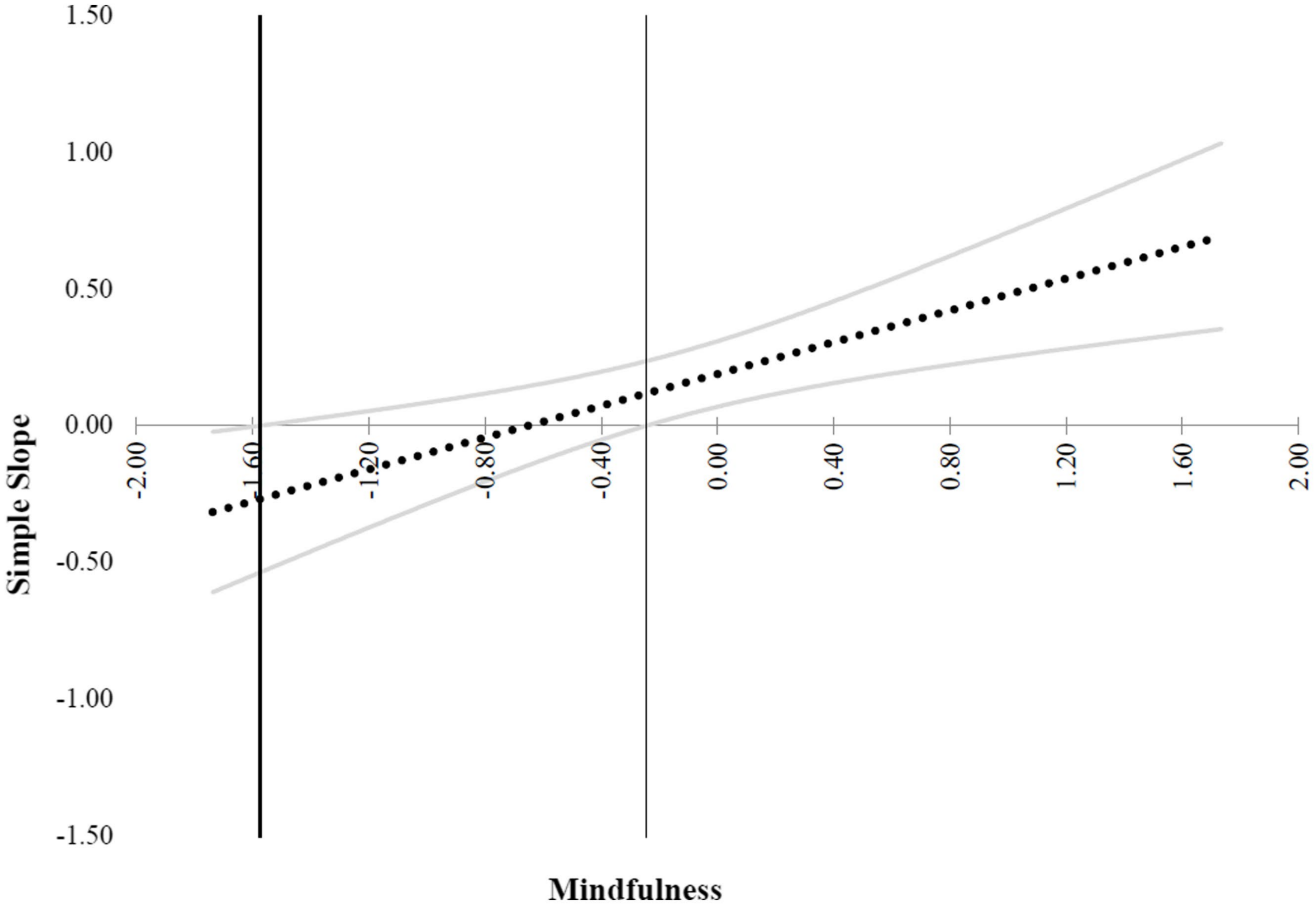
Regions of significance. Simple slope of workplace event criticality predicting work engagement by employee mindfulness. This figure presents the simple slope (*Y*-axis) across different scores of employee mindfulness (*X*-axis). Employee mindfulness was grand-mean-centered. Workplace event criticality positively predicts work engagement when the scores of employee mindfulness ≥ −0.2.

H3 predicted that the positive relationship between workplace event criticality and employee work engagement would become more salient when employee mindfulness increased. The results revealed that the interaction term was significantly and positively related to employee work engagement (*β* = 0.30, *p* < 0.01). Moreover, results of a simple slope test revealed that the positive effect of workplace event criticality on employee work engagement was significant for employees with a higher level of mindfulness (*simple slope* = 0.53, *t* = 3.73, *p* < 0.01), but not significant for employees with a lower level of mindfulness (*simple slope* = 0.13, *t* = 0.13, *n.s.*). Following the procedures outlined by Aiken et al. (1991) and Preacher et al. (2006), we plotted the interaction effect (see Figure 3) and regions of significance (see Figure 4). The figures showed that the relationship between workplace event criticality and employee work engagement is positive for employees with a higher level of mindfulness. H3 was thus supported.

H4 predicted employee mindfulness would intensify the indirect effects of workplace event criticality on employee proactive work behavior *via* employee work engagement. The results showed that the conditional indirect effect of workplace event criticality on proactive work behavior was positively significant for employees with a higher level of mindfulness (*indirect effect* = 0.09, 95% CI [0.01, 0.20]), but not significant for employees with a lower level of mindfulness (*indirect effect* = 0.003, 95% CI [−0.04, 0.05]). The difference of indirect effect was significant (*indirect effect difference* = 0.09, 95% CI [0.01, 0.20]), yielding support to H4.

Overall, *pseudo*-*R*^2^ indicates that 19% of the variances are in work engagement and 13% in proactive work behavior. We also rerun the path analyses, and our results are still robust when excluding the employee- and team-level control variables.

## Discussion and implications

In this study, drawing on event system theory and theory of engagement, we examined the role of workplace event criticality in stimulating employee proactivity. In a multi-wave and multi-source data collection of 179 employees from 55 supervisors, we found that employees who perceived workplace events as critical were more likely to increase their work engagement, stimulating proactive work behavior. Further, employee mindfulness strengthened the motivational potential of workplace event criticality in the workplace on employee proactive work behavior *via* work engagement.

### Theoretical implications

Despite the increasingly unavoidable influence of events in the workplace on employee proactivity, empirical research around their relationship and its underlying mechanism has been relatively sparse. Our event-oriented research advances this knowledge by unpacking the salient motivating role of workplace event criticality in employee work engagement and proactivity. It also increases our understanding by illustrating that employee mindfulness will amplify and intensify the motivational potential of workplace event criticality for work engagement.

The theoretical contributions of this study are 3-fold. First, we established a tentative explanatory model to illustrate the impact of critical workplace happenings on employees’ proactivity from an event-oriented theoretical perspective. The extant literature has well-documented a variety of feature-oriented contextual antecedents of employee proactivity, such as task autonomy and task significance (Parker et al., 2010). However, whether and how employees perceive workplace event criticality impacts their proactive behaviors remained unknown. Our findings complied with the existing literature that feature-oriented contextual factors promote employee proactivity. Specially, we found that workplace event criticality served as a stimulus for employee proactivity. This finding helped us gain valuable insight into the functioning of discrete and unpredictable workplace events concerning employee proactivity (Morgeson et al., 2015; Johns, 2017). Meanwhile, our study is also helpful for creating a complete nomological network of employee work behavior.

Second, this study provided robust empirical support for the impact of workplace event criticality on proactive work behavior by focusing on the mediating effect of work engagement. This result extended and advanced event system theory by considering work engagement’s vital role when employees react to critical environmental situations (Crant, 2000; Parker, 2000). Our results agreed with other recent research studying the motivating role of work context (e.g., task significance) on individual work engagement and how they increase specific positive behaviors, such as organizational citizenship behavior (Bakker and Schaufeli, 2008) or innovative behavior (Hakanen et al., 2008). However, these studies mainly focused on exploring the impacts of the stable and enduring aspects of the work context on work engagement, while our study focused on the impacts of discrete events on work engagement in the work context. Our findings indicated that discrete events, as critical discontinuous components of the work context, stimulated work engagement, thereby complementing the existing engagement studies. We found that critical workplace events can serve as a motivational force, which enriched the knowledge of event studies.

Third, we established a more fine-grained theoretical model by empirically examining the interactive effects of employees’ internal and relatively stable characteristics (i.e., mindfulness) and employees’ experienced external and unpredictable workplace characteristics (i.e., criticality). These factors interacted synergistically to stimulate work engagement and, subsequently, behavioral outcomes. Most event-oriented research has routinely taken a universal approach and focused on the main effects of event characteristics (e.g., Morgeson and DeRue, 2006). Few studies have taken a contingent approach and relied on individual characteristics to understand for which type of person the effect of workplace events will be amplified or buffered. Scholars called for more event-related research that should consider the integrative impacts of individual features (or traits) and event characteristics on employee behavior (e.g., Morgeson et al., 2015). This study echoed this call by examining the moderating role of mindfulness in transferring the effect of workplace event criticality to work engagement. The results showed that employees with a higher level of mindfulness experienced a significant effect of workplace event criticality on work engagement and proactive behaviors. However, when being less mindful, workplace event criticality did not appear to have a salient effect on employee work engagement and proactive behaviors. In this way, it contributes to the event-relevant and mindfulness literature. Our results confirmed the salutary effects of mindfulness in the workplace (Brown et al., 2007b) and the notion that individual mindfulness is beneficial for individuals coping with uncertainty and unpredictable environmental situations (Brown and Ryan, 2003), and complemented the existing literature that less mindful employees may not gain the above benefits when experiencing expectancies at workplace.

### Practical implications

This study also has several implications for management. First, our results indicate that workplace event criticality plays a critical role in generating positive outcomes through proactivity. This highlights the importance of firms leveraging events in the workplace to inspire these positive employee behaviors. Meanwhile, organizations should also provide employees with instant support and ongoing assistance with developmental feedback to foster proactivity in the presence of critical and unexpected situations (Morgeson, 2005; George and Zhou, 2007). For example, when introducing new equipment, employees might engage in proactive learning activities to improve their existing work methods and procedures when encouraged by their organization (Zhou and George, 2001).

Second, our findings suggest that workplace event criticality not only directly impacts employee proactivity but also indirectly through increased levels of work engagement. The findings demonstrate the motivational potential of workplace event criticality for work engagement. This allows firms to increase employee engagement by taking corresponding managerial measures when undergoing critical workplace events. For instance, when critical events in the workplace bring significant changes to employees’ job content (e.g., enterprise business transformation), organizations can initiate training and coaching programs to boost employee work engagement.

Third, our findings demonstrate the vital role of individual mindfulness. When experiencing critical workplace events, employees who are higher in mindfulness are more likely to concentrate and become engrossed in the work entirely, while less mindful employees lack work engagement and proactivity even confronting with unpredictable yet critical events at workplace. With the increasing happening of unexpected events in the workplace, managers can select and recruit mindful employees because this type of employees tends to keep engaged when facing unanticipated events. In addition, organizations can adopt training programs aimed at increasing the existing levels of employee mindfulness, for example, Mindfulness-Based Stress Reduction (MBSR; Kabat-Zinn, 1982) and Mindfulness-based Cognitive Therapy (MBCT; Hülsheger et al., 2013).

## Limitations and suggestions

Despite carrying theoretical and managerial implications, this study has several limitations that point to meaningful future research avenues. First, Morgeson et al. (2015) operationalized an event as having three features: a sense of disruption, novelty, and criticality. Criticality, novelty, and disruption represent different aspects of an event, and each characteristic can yield a strong enough outcome to induce behavioral change. However, research has recognized that event characteristics may create synergy and thus generate a more substantial effect on organizational entities than single characteristics alone (e.g., Morgeson et al., 2015). In this study, we focused on exploring the impact of workplace event criticality on employee proactive work behavior and controlled for event disruption. Future studies can elaborate on the potential interaction between event criticality and disruption and its synergistic effect on employee behavior.

Second, to make the study more fine-grained, we recommend that future studies employ qualitative research methods (e.g., in-depth interviews with employees) to shed additional light on how employees feel and react when confronted with critical events. Additionally, events are not always isolated but usually induce a series of secondary and derivative events. The exploration from “event” to “event chain” needs further development. Future research can consider the impact of events’ temporal and spatial attributes on employees’ attitudes and behaviors.

Finally, this study was based on data collected from service industry firms in China. To refine and test the generalizability of our research model, future research should extend it to other types of firms (e.g., manufacturing firms). Further, we asked the participants to recall the workplace events they experienced in the workplace over the past 1 or 2 months and evaluate the features of these events. Although this method has been commonly used in event-oriented research (e.g., Chen et al., 2021), retrospective measures have possible problems, such as existing recall bias (Rubin and Wenzel, 1996). For example, employees who are less engaged at work may be less likely to recall a true critical event exposure at workplace than those employees with high engagement. Consequently, the recall bias may cause the significant research findings based on retrospective measures interpreted by methodological artifact rather than theoretical explanations (Raphael, 1987). In this regard, it would be advisable for future studies to adopt other methods, such as conducting experiments to investigate the impacts of events happening on employees as they unfold. This could lead to a more accurate understanding of the influence of events and reduce recall bias (Ohly et al., 2010). Additionally, although the study analyzes longitudinal data, this cannot entirely rule out the possibility of reversed causality. We invite future studies to address this limitation with (quasi-)experimental studies.

## Data availability statement

The data that support the findings of this study are available from the corresponding author, upon reasonable request.

## Author contributions

YZ devised the project and the main conceptual ideas. LG worked out the methodology. LG and YF performed the analysis and interpretation of the data and contributed to the final version of the manuscript. YZ and LG drafted the manuscript. All authors contributed to the article and approved the submitted version.

## Conflict of interest

The authors declare that the research was conducted in the absence of any commercial or financial relationships that could be construed as a potential conflict of interest.

## Publisher’s note

All claims expressed in this article are solely those of the authors and do not necessarily represent those of their affiliated organizations, or those of the publisher, the editors and the reviewers. Any product that may be evaluated in this article, or claim that may be made by its manufacturer, is not guaranteed or endorsed by the publisher.
